# A Review of the Etiology and Epidemiology of Bladder Cancer: All You Need To Know

**DOI:** 10.7759/cureus.27330

**Published:** 2022-07-27

**Authors:** Sattam A Halaseh, Shahed Halaseh, Yaman Alali, Mohannad E Ashour, Mohammad J Alharayzah

**Affiliations:** 1 General and Colorectal Surgery, Torbay and South Devon NHS Foundation Trust, Torbay Hospital, Torquay, GBR; 2 Surgery, Jordan University Hospital, Amman, JOR; 3 Oncology, University of Nebraska Medical Center, Omaha, USA; 4 Urology, The Specialty Hospital, Amman, JOR; 5 Internal Medicine, Jordanian Royal Medical Services, Amman, JOR

**Keywords:** etiology, smoking tobacco, urothelial cell carcinoma, bladder ca, urinary bladder ca

## Abstract

Bladder cancer is any tumor that originates in the urinary bladder. It is the most prevalent tumor of the urinary system, with urothelial carcinoma being the most prevalent histologic subtype. It impacts both men and women. The development of bladder cancer was influenced by several risk factors, including advanced age, male sex, cigarette smoking, and occupational and environmental toxin exposure. Bladder tumors may manifest as gross or microscopic hematuria, which is assessed using cystoscopy, urine analysis, and other specialized tests. Due to the large number of cases related to environmental causes, bladder cancer is an appropriate target for public health preventative interventions. Cessation of smoking, adequate occupational safety procedures, diet, weight loss, and schistosomiasis prevention may mitigate the rising global incidence.

## Introduction and background

Bladder cancer, commonly referred to as urinary bladder cancer, is the tenth most prevalent cancer worldwide, and its prevalence is gradually increasing globally, particularly in industrialized nations. It continues to be the most prevalent malignancy of the urinary system [1]. The primary function of the bladder, a cavernous organ found in the lower abdomen, is to hold urine until micturition. Urothelial cells, which are specialized transitional epithelial cells that line the urinary bladder and urinary tract, accommodate the volume of urine generated by flattening under pressure. Additionally, the bladder is lined with smooth muscle that may relax to accept larger amounts and tighten to evacuate urine through the urethra [2]. The urothelial cells that line the bladder and urinary tract are always exposed to environmental chemicals that could cause mutations. The kidneys filter these chemicals out of the urine. It is not surprising that these urothelial cells, mostly found in the bladder, cause the majority of cancer cases, particularly in the developed world. Most bladder cancers may be dated directly to exposure to environmental and occupational toxins, with tobacco smoke being the most prevalent. Men's higher exposure to cigarette smoke and occupational hazards may help explain the fourfold gender disparity in bladder cancer incidence. Following tobacco use, the likelihood of bladder cancer is next only to the risk of lung cancer [1,3]. Unlike other malignancies, bladder cancer is seldom identified unintentionally during an autopsy. Eighty-five percent of patients with newly diagnosed bladder cancer have painless gross hematuria, and microscopic hematuria is present in virtually all patients [4]. Typically, hematuria is intermittent and may be associated with Valsalva movements. Therefore, a comprehensive evaluation of hematuria for bladder cancer consists of a focused history and physical examination in addition to diagnostic modalities such as bladder endoscopies, upper tract imaging, and urine culture. Although survival rates have improved as a result of earlier detection, robotic surgical methods, and the advent of immunotherapy, bladder cancer continues to be a major and growing contributor to the global cancer burden, particularly in wealthy nations [1]. On average, a patient's lifetime treatment costs for bladder cancer are higher than for any other malignancy. The overall treatment cost ranges from $129,000 to $251,000 per patient. It is anticipated that annual directed medical expenses will surpass $4 billion in the United States and €4.9 billion in the European Union (EU) [5]. Therefore, an improved understanding of the epidemiology and risk factors behind bladder cancer is essential for its prevention and reduction of its burden. This non-systematic literature review focuses on identifying the primary causes and risk factors of bladder cancer.

## Review

Epidemiology

Incidence

**Figure 1:**
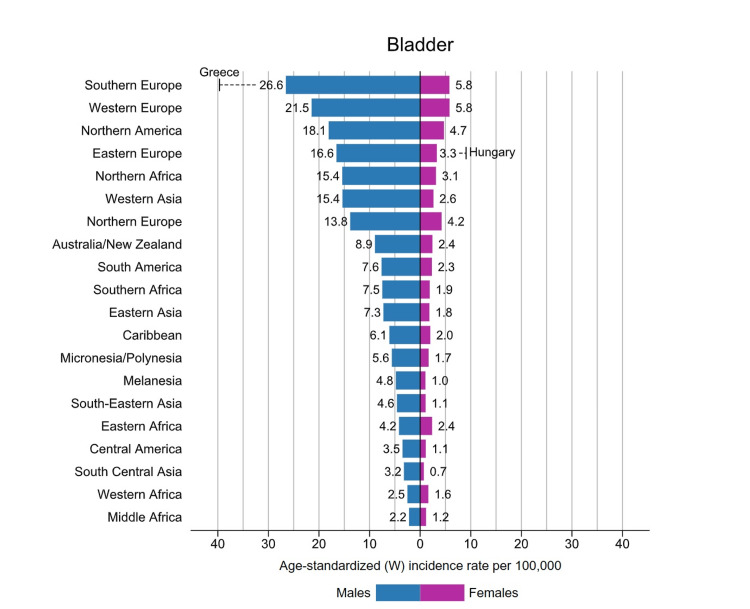
Bladder cancer incidence rates by region and gender in 2020. Source: reference [1].

As per Global Cancer Incidence, Mortality, and Prevalence (GLOBOCAN) data, an additional 573,000 cases of bladder cancer were identified in 2020. This accounts for around 3% of new cancer diagnoses. Most countries with a high incidence of bladder cancer are located in Southern and Western Europe and North America. Worldwide, Greece has the most significant incidence of bladder cancer among males, whereas Hungary has the most significant number of females. Southern Europe, where approximately 26.6 per 100,000 males and 5.8 per 100,000 females are diagnosed with bladder cancer each year, has the most significant incidence of bladder cancer among the global population [1], as shown in Figure 1. The territories with the lowest prevalence rate of bladder cancer are Middle Africa, South Central Asia, and Western Africa, which are largely comprised of countries with a human development index below the average, a consequence of less manufacturing exposure to chemicals and restricted access to tobacco [1].

In the United Kingdom, an estimated 12,400 instances of bladder cancer were diagnosed in 2020, constituting 2.7% of all cancer diagnoses. Thus, bladder cancer is the ninth most prevalent cancer in the United Kingdom [1]. Due to a more significant frequency of smoking and an older population, the prevalence rate of bladder cancer has continued to climb in several European countries, including Germany, and is projected to rise even more. Nevertheless, several nations have achieved substantial success in prevention, with New Zealand's incidence decreasing by roughly 10% over the previous decade [6]. Bladder cancer is nearly four times more prevalent in males than females, with incidence rates of 9.5 per 100,000 males and 2.4 per 100,000 females globally. Urinary bladder cancer is the sixth most prevalent and ninth most lethal tumor among males [1]. This disparity is most likely linked to gender disparities in cigarette use, which may also possibly clarify why cancer rates are growing among women in industrialized nations [1]. While bladder cancer is the tenth most prevalent malignancy in the world, it is the thirteenth most lethal, claiming an estimated 212,536 lives in 2020. This accounts for 2.1% of all cancer fatalities. Mortality rates follow incidence rates regarding the gender imbalance, with a mortality rate of 3.3 per 100,000 males, which is nearly four times higher than the mortality rate of 90 per 100,000 females globally (Table 1). Between birth and age 74, the cumulative probability of dying from bladder cancer is 0.3% for males and 0.08% for females. Geographical and temporal trends of bladder cancer incidence globally tend to mirror the prevalence of tobacco use. However, infection with Schistosoma haematobium and other chemicals exposure may be substantial contributors in some populations such as Egypt [1].

**Table 1:** Total global bladder cancer mortality in 2020. Source: reference [1].

Rank	Country	Number
	World	212,536
1	Egypt	6,170
2	Tunisia	822
3	Libya	242
4	Poland	5,026
5	Mali	426
6	Slovakia	629
7	Latvia	271
8	São Tomé and Príncipe	5
9	Algeria	1,861
10	Serbia	931

Due in part to breakthroughs in treatment (e.g., endoscopic resection, adjuvant instillation of chemotherapy, and IV immunotherapy), mortality rates have decreased primarily in the most developed settings, except for countries experiencing a rapid economic shift, such as those in Central and South America, some Central, Southern, and Eastern European countries, and the Baltic states [7,8]. According to data from the United Kingdom, 77.8% of males survived bladder cancer in their first year of diagnosis [9]. According to other statistics collected from the United States, the five-year survival rate was 77.1%. However, these percentages increase to nearly 96% for patients with an in-situ illness, reduce to nearly 69% for patients with local disease, and decrease even further to 36% and 4% for regional and metastatic diseases, respectively with a five-year survival [10].

Etiology

The wall of the urinary bladder consists of four components: mucosa, submucosa, muscularis, and serosa. The typical urothelium of the mucosa is composed of a seven-cell thick layer of stratified, non-squamous, homogeneous cells with big umbrella cells on top. Tumors arising from the urothelial cells compromise most bladder cancer, with a rough estimation of 90%. They are formally known as transitional cell carcinoma. Other non-urothelial bladder malignancies that can arise include squamous cell carcinoma (SCC), small cell carcinoma, adenocarcinoma, and other tumors with mixed histology [11,12]. Due to urothelial direct exposure, the urothelial subtype is strongly related to exposure to chemicals, such as workers exposed or tobacco smoking. On the other hand, squamous cell subclass instances are more prevalent in Africa, perhaps due to schistosomiasis, a protozoal illness that causes urinary bladder irritation and inflammation [13].

Risk factors

Tobacco

Tobacco is the primary recognized cause of bladder cancer, accounting for 30-40% of all cases of urothelial carcinoma and up to two-thirds of all bladder cancer. There are more than 1 billion active smokers worldwide, and smokers have two-to-three greater chances of developing bladder cancer [14]. Tobacco smoke has recognized carcinogens, including beta-naphthylamine and polycyclic aromatic hydrocarbons. The metabolism of these particles in the bladder and throughout the system results in the creation of DNA adducts and persistent genetic mutation. These mutations may activate oncogenes or inhibit tumor suppressor genes, encouraging carcinogenesis. It has been demonstrated that some hereditary genotypes associated with defective detoxification enzymes enhance the cancer predisposition in smokers [15]. Although cigarettes are the most prevalent tobacco product connected with bladder cancer incidence, pipe and cigar consumption have also been linked to the development of urothelial carcinoma [16]. One study demonstrated that quitting smoking reduces the risk of urothelial carcinoma. Those who have quit smoking for 1-3 years had a 2.6 relative risk compared to a 1.1 relative risk for those who have quit smoking for more than 15 years [17].

Gender

As noted previously, about three-quarters of bladder cancer cases occur in men with a greater incidence rate than in women [1]. Several theories have been suggested to explain the increase in male bladder cancer incidence. First, smoking is far more prevalent among males than females worldwide [18]. Although exposure to carcinogens may not account for variations between genders, the physiological breakdown of carcinogens could be distinct [19]. Enzymes involved in aromatic amine degradation and foreign material detoxification have been linked to carcinogen metabolism related to bladder cancer. It has been demonstrated that these enzymes are expressed differently in men and women [20]. In addition, variations in sex steroid synthesis and receptor expression underlie gender disparities. Age at menarche greater than 15 years, parity compared to nulliparous women, and use of estrogen or progestin medication have been linked to lower bladder cancer risk in women, suggesting that sex steroid exposure reduces bladder cancer risk [21]. From a tumor biology perspective, the androgen receptor (AR) has been linked to the genesis and progression of bladder cancer. AR expression appears to be downregulated in bladder cancer immunohistochemistry investigations, and this downregulation tends to increase with increasing tumor stage and grade [22]. In both males and females, current smokers develop bladder cancer six years earlier than current non-smokers.

Genetics and Hereditary

Although studies have failed to recognize important germline genetic factors underpinning sporadic bladder cancer, genome-wide correlation studies have identified several genetic loci having a small correlation with a genetic predisposition to bladder cancer [23]. Among these, N-acetyltransferase 2 (NAT2) and deletion of glutathione S-transferase (GSTM1) are both connected with the ability to metabolize aromatic amines and so play a significant role in the subgroup of individuals with environmental carcinogen exposure. Also, both appear to have a cancer-causing relationship with cigarette smoking [24]. Although first-degree relatives of bladder cancer patients have a twofold greater chance of getting urothelial bladder cancer, families at high risk for bladder cancer are extremely rare. The absence of a Mendelian inheritance pattern in hereditary bladder cancer renders traditional family-tree linkage analyses ineffective. Probability favors a complex explanation, with particular genes amplifying environmental stressors [25]. An increase in the incidence of urothelial and squamous bladder cancer has been related to mutations in the tumor-suppressor gene phosphatase and tensin homolog (PTEN) and the DNA mismatch repair gene MutS homolog 2 (MSH2), which are seen in Cowden and Lynch syndromes, respectively [26,27].

Age

One of the hallmarks of bladder cancer is its tendency to affect the elderly population. In the United States, more than 90% of people diagnosed with bladder cancer are over 55 years old, with a median age of diagnosis of 73 [28]. This suggests a disease progression that takes decades after contact with toxins to override cellular tumor-suppressor systems and results in carcinogenesis.

Occupational and Environmental Exposure

As with the skin and lungs, the bladder is a constantly exposed organ that is consequently susceptible to environmental toxins and inflammation. The second major avoidable risk factor for bladder cancer is occupational exposure to carcinogens such as aromatic amines (2-naphthylamine, 4-aminobiphenyl, and benzidine), which are responsible for 5-10% of all cases of bladder cancer [29]. These compounds are frequently used in manufacturing dyes, paints, metals, rubber, and petroleum goods. Working very closely with chemicals and dyes carries the highest lifetime risk. The vocations most at risk for exposure to aromatic amines include tobacco, dye, rubber professionals, hairdressers, painters, and leather workers. Those who deal with polycyclic aromatic hydrocarbons, such as chimney sweeps, nurses, alumni workers, petroleum workers, and sailors, are also in danger [29,30].

Infections and Pathogens

Multiple researchers have hypothesized that persistent or recurring bacterial UTIs may raise the chance of developing bladder cancer. Numerous epidemiologic studies suggest that chronic UTIs may be associated with a modestly increased risk of bladder cancer. However, these findings are flummoxed by chronic intermittent catheterization, chronic inflammatory bladder stone formation, and many other risk factors, such as smoking status and occupation [31]. Additionally, recurrent gonorrhea infections have been linked to the development of bladder cancer. One prospective research indicated that males with a history of gonorrhea have a roughly twofold increased chance of developing bladder cancer, and these tumors are more prone to being aggressive [32]. This is linked to the generation of carcinogens such as nitrosamines. Another important pathogen is schistosomiasis, a protozoan illness prevalent in as many as 76 poor nations, affecting as many as 236 million people. Schistosomes are blood flukes that parasitize both mammals and intermediate hosts (e.g., freshwater snails). However, Schistosoma haematobium (S. haematobium) is the only human schistosome associated with squamous cell bladder cancer. S. haematobium resides in the venules of the mammalian urinary bladder, where they produce inflammation and tissue fibrosis by laying their eggs. The process of SCC formation presumably includes a proinflammatory immune response of the T-helper cell-2 type [33]. In countries of the Middle East and Africa where schistosomiasis is predominant, SCC of the bladder is the second most prevalent form of cancer after hepatocellular carcinoma, which is also related to the disease [34].

Diet

Dietary variables have been extensively studied concerning bladder cancer incidence. Initial retrospective studies revealed a lower incidence of bladder cancer with higher water consumption [30]. In contrast, the European Prospective Investigation of Cancer and Nutrition (EPIC) found no association between total fluid consumption and bladder cancer risk. Nonetheless, there remains disagreement around fluid intake. In addition to fluid consumption, many carcinogens are absorbed through food and expelled in the urine, leading to significant contact with the urothelium and an elevated risk of bladder cancer. However, no solid evidence supports the intake of a particular diet or food group to reduce the risk of bladder cancer. For example, meat consumption has been linked to a higher risk of developing different types of cancer. However, among more than half a million participants in the EPIC trial, the red meat diet was not linked to bladder cancer risk [35].

Medical Illness

Medical disorders may raise the risk of bladder cancer either directly or indirectly through the toxicity of therapy. Typically, persistent inflammation and the formation of keratinizing squamous metaplasia drive direct carcinogenesis. In addition, it has been hypothesized that bladder stones, urinary outflow obstruction, repeated UTIs, and irritation from direct catheter damage contribute to the development of metaplasia and increase the risk of SCC of the bladder [36]. Bladder cancer is often caused by unforeseen side effects of medicinal therapy. One of those drugs includes pioglitazone. It is a thiazolidinedione-class antidiabetic medication that reduces glucose levels in individuals with non-insulin-dependent diabetes. In 2005, the PROactive randomized trial reported the unexpected discovery that the pioglitazone group had more bladder cancer cases than the placebo group [37]. Another important risk factor is chemotherapy. It eliminates cancerous cells by producing DNA damage and cell death; nevertheless, chemotherapy might produce dysregulation in normal cells in organs with a fast cell turnover. Cyclophosphamide is the only chemotherapy that has been proved to induce bladder cancer. Phosphoramide mustard is the major mutagenic metabolite that causes bladder cancer caused by cyclophosphamide. Patients who had cyclophosphamide therapy have a 4.5-fold increased risk of developing bladder cancer, which appears to be dose-dependent and is greatest among those who received more than 20 grams [38]. The genesis of urothelial carcinoma following radiation therapy does not seem to be age-dependent; nevertheless, the estimated latency time is 15-30 years [39]. External beam radiation treatment for cervical cancer is related to a two- to fourfold increased incidence of subsequent bladder cancer compared to the non-irradiated group [40].

BMI

A growing body of data indicates that obesity, defined as BMI above 30 kg/m2, may be carcinogenic, and multiple cancer types correlate with increased BMI. Multiple studies have demonstrated that increasing BMI is a significant risk factor for bladder tumor progression [41]. One reason for this relationship is the high correlation between smoking and obesity; nevertheless, even after correcting the smoking status, BMI remains related to bladder cancer. According to research on how obesity may promote carcinogenesis, obesity increases insulin production, which may stimulate tumor growth. Expansion of adipose tissue increases the synthesis of proinflammatory proteins and cytokines (such as tumor necrosis factor and interleukin-6) while decreasing the synthesis of the protein adiponectin. These metabolic irregularities result in hyperinsulinemia and insulin resistance [42]. To control normal glucose levels, these alterations might cause pancreatic beta cells to increase insulin synthesis, resulting in hyperinsulinemia. Hyperinsulinemia augments the expression of insulin-like growth factor 1 [43], which in turn causes an imbalance in cell proliferation, death, and angiogenesis [42], hence impacting the development of bladder cancer [44].

Lowering risk and prevention

According to recent studies and analyses, nearly 82% of bladder cancer diagnoses from the last 20 years might be related to known preventive factors. Only 7% of bladder cancer occurrences are anticipated to be caused by inherited genetic factors [45]. Bladder cancer is an ideal target for public health preventative measures due to the high proportion of cases attributed to established environmental factors. Cigarette smoking is by far the largest potential risk for bladder cancer in the industrialized world, accounting for up to two-thirds of all cases. It has been established that quitting smoking reduces the incidence of cancer. Similarly, second-hand smoking exposure raises danger and must be eliminated [46]. The second highest avoidable health risk for bladder cancer is occupational exposure. Those in the manufacturing, transportation, firefighting, and hair styling industries should take precautions to minimize chemical exposure through aerosols and touch [30]. The degree to which a fruit-and-vegetable-rich diet can mitigate bladder cancer is still up for debate. Physical exercise has been proven to have a minor protective impact against bladder cancer irrespective of smoking and BMI. Its benefit may be augmented if incorporated into a weight loss program [47]. In endemic places, sanitizing, drinking water, showering, and avoiding swimming and wading in freshwater might substantially reduce the incidence of schistosomiasis [48]. The mass use of the anthelmintic drug praziquantel might control the illness and substantially reduce the chance of contracting bladder cancer [49]. Regular screening for bladder cancer in the general population is not recommended by any major professional organizations at this time. In individuals who are at medium risk, no screening test has been demonstrated to reduce the chance of dying from bladder cancer. People at a very high risk of developing bladder cancer, such as those who have had the disease in the past, those who have specific congenital abnormalities in the bladder, and those who have been subjected to certain toxins at work, may be advised to undergo testing for the disease [40,50]. These tests may include a urine analysis, cytology, a urine test for tumor indicators such as bladder tumor-associated antigen (BTA), UroVysion, ImmunoCyt, and a cystoscopy of the bladder [51].

## Conclusions

Bladder cancer is one of the most prevalent cancers in the world. Its incidence is on the rise, particularly in Europe and other industrialized nations, although deaths are down globally due to increased prevention, early detection, and treatment. The prognosis for those with metastatic disease is not good. Tobacco smoking is the largest contributing factor to the urothelial subtype, which accounts for 90% of all cases. Two-thirds of all bladder cancer occurrences are attributable to smoking, which increases the likelihood of the illness by two to three times. In contrast, SCC of the bladder, which represents a minority of occurrences, is prevalent in Africa and the Middle East and is strongly linked to schistosomiasis, a protozoal infection. Occupational and environmental interaction with carcinogenic substances is the second largest risk factor behind smoking. Approximately 82% of bladder cancer cases are attributable to modifiable risk factors. Therefore, prevention initiatives, such as smoking cessation, appropriate workplace safety practices, nutrition, weight reduction, and schistosomiasis prevention, might considerably alleviate the increasing global incidence of bladder cancer.
